# From Waste to Defense: Agro-Industrial Byproducts as Sources of Biopesticides and Bioelicitors for Crop Protection

**DOI:** 10.1021/acs.jafc.5c13266

**Published:** 2026-03-25

**Authors:** Marco Greco, Giulia Caminada, Daniele Coculo, Vincenzo Lionetti

**Affiliations:** † Institute for Sustainable Plant Protection, National Research Council (IPSP-CNR), Bari, Apulia 70126, Italy; ‡ Università degli Studi di Roma La Sapienza, Biologia e Biotecnologie “Charles Darwin”, Rome, Lazio 00185, Italy

**Keywords:** plant immune response, induced resistance, antimicrobial activity, insecticidal activity, cell wall oligosaccharides, proteins, essential oils, phenolic compounds, glucosinolates, glycoalkaloids, plant protection products, circular bioeconomy

## Abstract

The intensification of agro-industrial production has led to a heavy reliance on chemical pesticides, raising significant environmental and health concerns. Sustainable alternatives can be found in the plant kingdom, which employs complex defense mechanisms against pests and phytopathogens, including the biosynthesis and release of antimicrobial and immune elicitor compounds. However, the increasing demand for plant-based foods limits their extraction and commercial use. Agro-industrial factories generate large amounts of underutilized plant-based waste, whose management poses significant challenges. Agro-industrial byproducts accumulate high concentrations of bioactive molecules that are retrieved through green extraction methods that show promising results for controlling pests and phytopathogens. The repurposing of plant-based agro-industrial byproducts for biopesticides and vaccines for plant development can offer crucial help in the implementation of the circular economy, resilient agricultural systems, and sustainable crop protection.

## Graphical abstract

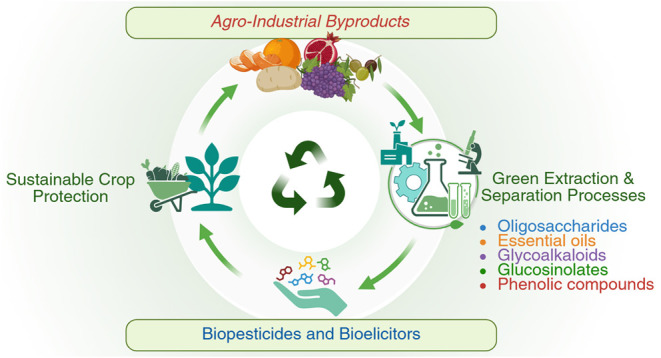

## 1 Introduction

The global demand for agricultural products has surged in recent years to meet the needs of the growing world population that is projected to reach approximately 9.7 billion by 2050.^1^ Rising demands for food and agricultural production have driven a shift from traditional farming practices to intensive cultivation systems that heavily rely on synthetic inputs.^2^ Over 3 billion kilograms of chemical pesticides, including insecticides, nematicides, fungicides, and bactericides, are used worldwide each year to control pests and phytopathogens, with a purchase price of nearly $40 billion annually.^3^ Without pesticide application, estimated production losses are generally 78% for fruits, 54% for vegetables, and 32% for cereals, leading to substantial economic losses that amount to several billion dollars annually.^4^ Besides the production losses, the use of pesticides is required to reduce the occurrence of mycotoxigenic fungi in food and feed that can pose a serious threat to human health and livestock.^5^

The increasing use of pesticides is further promoted by changes in the climate, which expose plants to environmental stresses that weaken their natural defense mechanisms and, consequently, intensify pest infestations and plant diseases by expanding their geographic distribution. However, extensive pesticide application raises major concerns due to environmental persistence and contamination of air, soil, and water, threatening plant and animal life. Moreover, pesticides negatively affect human health, particularly agricultural workers and consumers exposed through food residues.^6^ Reported toxic effects include neurotoxicity, cancer, asthma, reproductive disorders, cardiovascular diseases, and diabetes.^7^ These risks have led to the restriction or banning of several compounds, emphasizing the need for alternative strategies.^8^ Consequently, agricultural and environmental policies, including the European strategies “Green Deal” and “Farm-to-Fork”, promote the replacement of chemical pesticides with natural and organic products.^9^

Plants produce a wide range of antimicrobial compounds, classified as phytoanticipins and phytoalexins according to their biosynthesis and accumulation.^10^ Phytoanticipins are constitutively synthesized and stored in specialized tissues, whereas phytoalexins are produced *de novo* following pathogen attack. Both play a central role in plant defense and display antioxidant, insecticidal, antibacterial, and antifungal activities against numerous pests and phytopathogens.^11,12^ They are synthesized as secondary metabolites that can be classified in chemically diverse groups such as steroidal glycoalkaloids (SG), essential oils (EO), cyanogenic glycosides, phenolic compounds (PC), glucosinolates (GLS), and terpenoids.^13^ The secondary metabolite production can be driven by perception of diverse elicitors through specific plasma membrane-resident Pattern Recognition Receptors (PRRs), which activate innate host defense mechanisms.^14,15^ A key class of plant defense elicitors is Damage-Associated Molecular Patterns (DAMPs), encompassing oligosaccharides, proteins, nucleotides, DNA, and amino acids.^16^

Oligosaccharides are a significant group originating from the breakdown of the plant Cell Wall (CW), a complex and dynamic biological network composed of proteins, PC, minerals, water, and interacting polysaccharides such as cellulose, hemicellulose, and pectin.^17^ During plant-pathogen interactions, oligosaccharide DAMPs are released by microbial CW-degrading enzymes (CWDEs) to break down CW structural barriers and enter plant cells.^18,19^ Pectin represents a major source of CW-derived DAMPs and consists of galacturonic acid-rich polysaccharides, including homogalacturonan (HG), rhamnogalacturonan (RG) I and II, and xylogalacturonan.^20–22^ Important pectin-derived DAMPs are the well-characterized Oligogalacturonides (OG), which are HG fragments released by microbial polygalacturonases, and RG-I oligosaccharides generated by the action of RG-I lyases and RG-I hydrolases.^23–25^ Additional DAMPs originate from hemicellulose fragments and cellulose-derived molecules such as cellobiose and cellodextrins.^26,27^ During infection, plants also release apoplastic peptides known as phytocytokines, which are produced as inactive precursors and processed into active forms by specific proteases.^28,29^ These peptides are perceived by PRRs and contribute to immune activation and amplification of DAMP signaling.^30,31^ Plants further detect Microbe-Associated Molecular Patterns (MAMPs), including peptides derived from Flagellin (Flg), Elongation Factor Tu (EF-Tu), and cold shock proteins.^29,32^ The perception of DAMPs, phytocytokines, and MAMPs activates Pattern-Triggered Immunity (PTI), characterized by early signaling events such as Ca^2+^ influx, Reactive Oxygen Species (ROS) production, and activation of protein kinases.^33^ These processes induce transcriptional reprogramming, CW reinforcement, antimicrobial compound synthesis, and phytohormone signaling, mainly involving jasmonic acid, salicylic acid, and ethylene.^34,35^

Successful early defense leads to induced resistance through defense priming, a form of stress memory that enhances future responses.^14^ Jasmonic acid and ethylene signaling pathways regulate induced systemic resistance, and salicylic acid pathways induce systemic acquired resistance.^36^ Priming involves the accumulation of PRRs, Mitogen-activated protein kinase (MAPKs), their transcripts, and chromatin modifications that amplify immune signaling.^37^ This phenomenon shows functional similarities to immune memory in animals, as both kingdoms exhibit enhanced preparedness following initial exposure, mediated by epigenetic and transcriptomic reprogramming, despite the lack of specialized immune cells in plants.^38^

These natural compounds represent promising candidates for new biopesticide formulations as eco-friendly and cost-effective alternatives to synthetic pesticides. The global biopesticide market, valued at USD 7.54 billion, is projected to reach USD 28.61 billion by 2032, with plant-based products representing a substantial fraction.^39^ Although several commercial plant-derived biopesticides exist (Table 1), they still account for only about 5% of the pesticide market.

**Table 1:** Examples of Commercial Plant-Extract-Based Formulations Used for Insect and Mite Control

**active molecules**	**plant source**	**target**	**ref**
azadirachtin	*Azadirachta indica*	insects, mites	^235,236^
pyrethrin	*Tanacetum cinerariifolium*	insects	^237^
limonene	*Citrus* Sp.	insects	^238^
geraniol oil, peppermint oil	*Mentha* × *Piperita*, *Cymbopogon* Sp.	insects, mites	^239^
rosemary oil, clove oil, thyme oil, peppermint oils	*Rosmarinus officinalis*, *Syzygium aromaticum*, *Thymus vulgaris*, *Mentha* × *Piperita*	insects, mites	^239^
oleoresin, garlic oil, soybean oil	*Capsicum* Sp., *Allium sativum*, *Glycine max*	insects, mites	^240^
matrine	*Sophora flavescens*	insects	^241^
veratrine	*Veratrum nigrum*	insects	^242^
acetogenin annonin	*Annona squamosa*	insects	^236,243^
karanjin	*Derris indica*	insects, mites	^236^

Plant bioactive molecules offer advantages such as environmental compatibility, low toxicity to nontarget organisms, and diverse modes of action that limit the development of resistance in pathogens.^40^ However, their application is constrained by competition with food production.^41^ Consequently, alternative sources such as agro-industrial byproducts (AIB) are gaining attention.^42^

The agro-industrial sector generates approximately 140 billion tons of AIB annually, largely of plant origin.^43^ Most AIB are not productively utilized and are instead disposed of through burning, dumping, or landfilling, posing significant environmental, economic, and social challenges.^44^ European Union Directive 2018/851 identifies their reduction and valorization as priorities within circular economy strategies.^45^ Upcycling AIB into sources of bioactive molecules could support pest control, reduce chemical pesticide use, mitigate the environmental impact, promoting sustainable agriculture.^46^ Bioactive molecules can accumulate at higher amounts in AIB with respect to the original plant material due to their concentration during industrial processing.^47^ Despite their potential benefits, the agricultural application of AIB-derived bioactive compounds remains limited. This review, therefore, examines the environmental impact of plant-based AIB, their bioactive content, and the extraction and fractionation strategies used to recover these biomolecules as well as their potential use as antimicrobial and insecticidal agents as well as vaccines for crop protection.

## 2 Agro-Industrial Byproducts are Mines of Bioactive Molecules

Plant-based AIB can be categorized into agricultural and industrial wastes.^48^ Agricultural wastes include field residues (e.g., leaves, stems, and bunches) and process residues such as husks, roots, and seeds generated during raw material processing, whereas industrial wastes comprise organic residues and effluents from food industries, including peels, pomace, grounds, oils, and molasses. The large volumes and complex composition of AIB create major challenges due to high disposal costs and frequent improper management, often resulting in incineration or landfilling with serious environmental consequences.^49^ The main component of AIB is lignocellulosic biomass, which consists of cellulose, hemicellulose, and lignin. Globally, more than 2 billion tons of lignocellulose-rich wastes are burned every year, releasing large amounts of dust, smoke, and toxic gases such as SO_2_, and greenhouse gases including CO_2_, CH_4_, and N_2_O, thereby contributing to air pollution, respiratory diseases, and climate change.^50^ These gases can generate acidic compounds that lead to acid rain, further damaging ecosystems.^51^ AIB burning can also emit carcinogenic substances, including furans, dioxins, and polycyclic aromatic hydrocarbons, which persist in the environment and bioaccumulate in food chains, posing public health risks.^52^ Uncontrolled landfilling of AIB is similarly problematic, as their high moisture content and biodegradable organic matter promote microbial growth, greenhouse gas emissions, and bacterial contamination.^53^ Soil quality may also be impaired by the accumulation of secondary metabolites with phytotoxic and antimicrobial effects. Accordingly, European Union Directive 2018/851 classifies olive mill wastes as highly polluting due to their high phenolic content.^45^ Among olive mill wastes, olive mill wastewater can possess a phenolic content ranging from 2 to 10 g/L, and olive leaf can contain a phenolic amount of about 60–120 mg of gallic acid equivalents per g of dry weight.^54^ PC compounds contain one or more hydroxylated aromatic rings. Their discard on land was reported to cause the inhibition of seed germination and plant growth, the degradation of soil structure, the reduction of microbial diversity in the soil, with eventual soil fertility decrease.^55^ Grape pomace, another phenolic-rich byproduct (10–30 mg gallic acid equivalents per g), inhibits nitrogen mineralization and decreases nitrogen availability to plants.^56,57^ AIB are known to be enriched in several bioactive molecules such as SG, EO, and GLS.^58^ Although direct links with toxicity are not always established, high concentrations may cause ecotoxicological risks. Alkaloids are derived from amino acids and contain at least one nitrogen atom within a heterocyclic structure. Toxic pyrrolizidine alkaloids can accumulate in AIB from Boraginaceae oil production or through coharvesting of alkaloid-producing weeds with crop plants, contaminating residues that may enter soils and crops and raise food safety concerns.^59–61^ Consumption of alkaloid-contaminated food can lead to hepatotoxicity, musculoskeletal disorders, and death.^62,63^

EO-rich AIB originate mainly from citrus processing residues (such as peels and pomace) or discarded aromatic plants, mainly from Lamiaceae species such as thyme and oregano.^64^ EO is a complex mixture of numerous low-molecular-weight molecules, primarily composed of terpenoids and phenylpropanoids. While EO generally show low environmental persistence, their thymol- and carvacrol-rich fractions can be toxic to the honeybee *Apis mellifera*, reducing survival and mobility and affecting the nervous system.^65,66^ GLS consist of a β-d-glucosyl residue linked by a sulfur atom to an invariant *cis*-*N*-hydroxyminosulfate ester, and a variable side chain derived from a modified amino acid.^67^ GLS, common in Brassicaceae processing residues, can negatively affect soil microbial communities, nitrifying bacteria, and plant growth, altering nutrient cycling.^68^ These environmental risks increase remediation costs and burden agro-industrial economies, highlighting the need for circular bioeconomy strategies to reduce the carbon footprint and upcycle AIB. Targeted extraction of bioactive compounds could lower pollutant loads while simultaneously generating high-value products.

## 3 Sustainable Extraction And Separation Technologies: Advancements Beyond Conventional Methods

Recovery of these compounds is challenging because extraction efficiency depends on both AIB properties and target molecule chemistry, while also requiring sustainability and cost-effectiveness.^43,69^ Traditional extraction methods are time-consuming, solvent-intensive, and environmentally harmful.^70^ Traditional extraction methods, such as maceration, Soxhlet extraction, and solid–liquid extraction using aqueous, alcoholic, or hydroalcoholic solvent systems, as well as hydrodistillation, remain widely used to recover bioactive compounds from plant materials, but they often require large amounts of solvents and energy and long processing times and offer limited selectivity. In recent years, increasing attention has been devoted to more sustainable alternatives, which can be broadly divided into advanced extraction techniques and downstream separation processes.^71^ Modern extraction approaches, including ultrasound-assisted extraction, microwave-assisted extraction, and subcritical water extraction, improve mass transfer and extraction efficiency while reducing the solvent consumption and thermal degradation of sensitive compounds. In addition, physical pretreatments such as acid hydrolysis and steam explosion have been applied to lignocellulosic agro-industrial byproducts to enhance the release of biomolecules and improve the extractability. An additional contribution to sustainability derives from the increasing use of green solvents, including water, ethanol, supercritical CO_2_, and deep eutectic solvents. Solvent-free mechanical processes, such as cold pressing, are also applied for the recovery of essential oils from citrus byproducts, further reducing the environmental impact. Among these, natural deep eutectic solvents composed of naturally occurring primary metabolites, such as organic acids, sugars, amino acids, and choline derivatives, are attracting particular interest due to their low toxicity, biodegradability, tunable polarity, and high solubilization capacity for diverse classes of bioactive compounds.

These methods can be effectively combined with separation technologies. An innovative solvent-free method based on tangential flow membrane filtration (also referred to as cross-flow filtration) was developed.^72^ This membrane technology, integrating sequential microfiltration, ultrafiltration, nanofiltration, and reverse osmosis steps, is defined as Best Available Technology and recognized by the Environmental Protection Agency due to its promising industrial applications.^73^ Tangential flow membrane filtration allows for the concentration and partial purification of target molecules from complex extracts using mild conditions and minimal organic solvents. Similar membrane-based approaches are also applied after aqueous or hydroalcoholic extraction to improve the fractionation efficiency and reduce solvent use. This approach can be complemented by downstream steps to further improve extract purity and consistency, such as sequential filtration, adsorption on polymeric resins for polyphenol recovery, and solvent recycling coupled with chromatographic purification.^74^ In some cases, low-energy physical separation steps, including centrifugation, are employed prior to or in combination with filtration to remove suspended solids and stabilize complex extracts. Selected AIB-derived fractions can also undergo functionalization or chemical modification to enhance their biological activity and consistency, as reported for specific plant defense inducers.

## 4 Agro-Industrial Byproducts as Sources of Molecules with Pesticidal Activity

Plant-based biopesticides, referred to as phytopesticides, can derive their bioactive ingredients from a wide range of plant families, including *Amaryllidacea, Apiaceae, Asteraceae, Brassicaceae, Cucurbitaceae, Fabaceae, Lamiaceae, Lauraceae, Malvaceae, Meliaceae, Myrtaceae, Oleaceae, Piperaceae, Poaceae, Rutaceae, Solanaceae*, and *Vitaceae*.^75,76^ Compounds are generally regarded as phytopesticides when they disrupt cell membranes, inhibit key enzymes and physiological processes, or target pest nervous systems, thereby impairing growth, development, and reproduction.^77^ Several plant biomasses are the object of agro-industrial processes that produce plant-derived AIB rich in phytopesticidial molecules.^78^ These compounds are naturally produced by plants as part of their defense system to protect tissues directly from pathogens and herbivores. The following paragraphs present examples of natural compounds extracted from different AIB that have been investigated for their biopesticide activities against pathogens (Table 2).

**Table 2:** Agro-Industrial Byproduct Extracts with Antimicrobial and Insecticidal Activity Against Plant Pathogens and Pests^a^

**byproducts**	**species**	**extraction**	**fractionation**	**composition**	**target organisms**	**refs**
olive pomace	*Olea europaea*	hydroalcoholic	filtration	N.R.	*Alternaria alternata*, *Cladosporium* Sp., *Fusarium proliferatum*	^88^
		hydroalcoholic	filtration	N.R.	*Alternaria solani*, *Botrytis cinerea*, *Fusarium culmorum*	^87^
		N.R.	tangential flow membrane filtration	77% hydroxytyrosol	*Pectobacterium carotovorum*, *Pseudomonas syringae*, *Xylella fastidiosa*, *B. cinerea*, *Collectotrichum graminicola*, *Fusarium graminearum*	^89^
				18.5% tyrosol		
olive mill wastewater		organic solvents	resin-based	52.7% hydroxytyrosol	*A. solani*, *P. syringae*, *Verticillium dahliae*, *Xanthomonas campestris*	^244^
					*P. carotovorum*	^227^
					*V. dahliae*	^228^
		aqueous	tangential flow membrane filtration	64.7% hydroxytyrosol	*V. dahliae*	^91^
				25.4% verbascoside	*Agrobacterium tumefaciens*, *Pseudomonas savastanoi*	^90^
				57–74.4% Oleuropein, 10.8–33.5% verbascoside	*X. fastidiosa*	^95^
olive leaves		aqueous/ultrasonic	filtration	45.6% leuropein, 12.5% catechol	*X. fastidiosa*	^96^
pomegranate fruit peel	*Punica granatum*	aqueous	filtration	N.R.	*Monilinia fructigena*, *Monilinia laxa*	^245^
			filtration	punicalagin	*B. cinerea*	^102^
		aqueous	filtration		*A. alternata* *, Fusarium* Spp., *Stemphylium botryosum*,	^101^
		organic solvents/ultrasonic	filtration		*Fusarium oxysporum*	^103^
		alcoholic	filtration		*B. cinerea*, *M. laxa*, *Penicillium digitatum*, *Penicillium expansum*, *Penicillium italicum*	^104,106,246^
		organic solvents	filtration	45.7–58.4% punicalagin	*B. cinerea*, *P. italicum*, *P. digitatum*, *Rhizopus stolonifer*	^105,247,248^
		organic solvents	filtration	N.R.	*Aspergillus flavus*	^107^
		organic solvents	filtration	40.5% chlorogenic acid, 29% catechin	*Fusarium sambucinum*	^108^
		hydroalcoholic	resin-based	68.3% punicalagin	*Ralstonia solanacearum*	^109^
		alcoholic	filtration	46% ellagic acid	*P. syringae* Pv. *Tomato*	^110^
		hydroalcoholic/ultrasonic	filtration	punicalin and gallic acid	*X. campestris* Pv. *Campestris*	^111^
grape stem	*Vitis vinifera*	organic solvents	filtration	N.R.	*A. flavus*, *Aspergillus fumigatus*, *Aspergillus niger*, *Penicillium chrysogenum*, *P. italicum*, *P. expansum*	^120^
		hydroalcoholic/ultrasonic	filtration	p-coumaric acid, ferulic acid	*Aspergillus carbonarius*	^121^
grape seeds and skins		acid hydrolysis/steam explosion	filtration	gallic acid, hydroxybenzoic acid, p-coumaric acid, vanillic acid	*F. oxysporum*	^123^
grape skins		organic solvents	filtration	N.R.	*P. chrysogenum*, *P. expansum*, *A. niger*, *Aspergillus versicolor*	^249^
grape pomace		hydroalcoholic	N.R.	malvidin	*P. syringae* Pv. *Actinidiae*	^124^
		organic solvents	N.R.	catechin, epicatechin, miquelianin, phloroglucinic acid, and procyanidin	*Phytophthora cinnamomi*	^125^
		supercritical CO_2_	N.R.	gallic acid, chlorogenic acid, kaempferol, ferulic acid, quercetin	*A. niger*	^126^
		hydroalcoholic/ultrasonic	Filtration	oenin, myrtillin, kuromanin	*A. flavus*, *A. carbonarius*, *F. graminearum*	^127^
potato tuber peel	*Solanum tuberosum*	organic solvents	Filtration	α-solanine, α-chaconine	*Fusarium solani*	^137^
		hydroalcoholic/ultrasonic	Filtration		*Fusarium sulphureum*	^139^
		N.R.	Chromatography/Filtration	α-solanine	*B. cinerea*	^142^
citrus fruit peel	*Citrus paradisi*, *Citrus reticulata*	organic solvents/soxhlet apparatus	Filtration	limonene	*Rhyzopertha dominica*	^250^
	*Citrus aurantium*, *Citrus limon*	cold pressing	N.R.	N.R.	*Colletotrichum okinawense*	^151^
	*C. limon*, *C. reticulata*, *C. paradisi*, *Citrus sinensis*			N.R.	*A. niger*, *A. flavus*, *P. chrysogenum*, *Penicillium verrucosum*	^152^
	*C. reticulata*, *Citrus grandis*, *Citrus aurantifolia*			N.R.	*Mucor hiemalis*, *P. expansum*, *F. proliferatum*	^153^
	*C. reticulata*			N.R.	*P. chrysogenum*	^154^
	*C. aurantium*			N.R.	*F. graminearum*, *F. culmorum*	^156,251^
	*C. grandis*, *C. limon*			N.R.	*Fusarium*	^157^
	*C. aurantium*	hydrodistillation		linalyl acetate	*Penicillium crustosum*, *Penicillium citrinum*, *P. expansum*	^155^
	*C. limon*			citral	*Fusarium verticillioides*, *F. proliferatum*	^158^
	*C. sinensis*		N.R.	N.R.	*Callosobruchus maculatus*	^252^
					*R. dominica*	
					*Tribolium confusum*	
	*C. reticulata* × *C. sinensis*, *Citrus clementina* × *C. reticulata*, *C. reticulata*			N.R.	*A. alternata* F. Sp. *Citri*	^164^
	*C. sinensis*, *Citrus maxima*			N.R.	*Aspergillus Terreus*, *A. alternata*, *Aspergillu niger*, *F. oxysporum*, *Helminthosporium oryzae*	^161,253^
	*C. sinensis*			limonene	*A. alternata*, *Alternaria mali*, *A. niger*, *B. cinerea*, *Cladosporium cladosporioides*, *Cladosporium fulvum*, *P. chrysogenum*, *P. expansum*, *Botryodiplodia theobromae*, *Myrothecium roridum*	^160,254^
	*C. reticulata*	organic solvent/distillation	N.R.	N.R.	*P. expansum*, *Penicillium sclerotiorum*, *P. digitatum*, *P. italicum*	^162^
	*C. sinensis*			N.R.	*A. niger*	^255^
	*C. limon*	hydrodistillation	N.R		*P. italicum*	^256^
	*C. aurantium*, *Citrus aurantofolia*, *Citrus limonium*, *Citrus latifolia*, *C. reticulata*, *C. sinensis*, *C. paradisi*			N.R.	*C. maculatus*	^168–170^
	*C. sinensis*			N.R.	*Zabrotes subfasciatus*, *Sitophilus zeamais*, *Sitophilus oryzae*, *Tribolium castaneum*	^171,257–259^
	*C. latifolia*, *C. maxima*, *Citrus Unshiu*, *C. reticulata*, *C. limon*, *Citrus lemon*, *C. reticulata* × *C. paradisi*, *C. aurantifolia*			N.R.	*Phaeoramularia angolensis*	^163^
	*C. aurantium*			linalyl acetate	*Xanthomonas citri* Subsp. *Citri*	^173^
	*C. aurantium* Var. *Amara*			linalool	*Erwinia amylovora*, *P. savastanoi*, *Xanthomonas vesicatoria*, *Allorhizobium vitis*	^175^
spent hop	*Humulus lupulus*	hydroalcoholic/ultrasonic	Filtration	myrcene, A-caryophyllene, B-caryophyllene, cadinene, humulene epoxide	*F. graminearum*, *A. carbonarius*, *A. alternata*	^127^
defatted seed meal	*Brassica carinata*	N.R.	N.R.	allyl isothiocyanate	*Monilia laxa*, *B. cinerea*, *Tetranychus urticae*, *Podosphaera xanthii*	^183,185,188,189^
	*Brassica juncea*				*P. expansum*	^184^
	*B. juncea*, *Sinapis alba*				*F. oxysporum*	^192^
	*Brassica rapa*			butenyl isothiocyanate	*M. laxa*	^183^
	*B. carinata*			isothiocyanate	*Erysiphe betae*, *Erysiphe cichoracearum*	^186^
	*B. carinata*				*Fusicladium oleagineum*	^190^
	*B. juncea*				*Melolontha* Spp.	^260^

a N.R.: not reported, ×: interspecific hybridization.

Their proposed mechanisms of action are illustrated in Figure 1.

**Figure 1:**
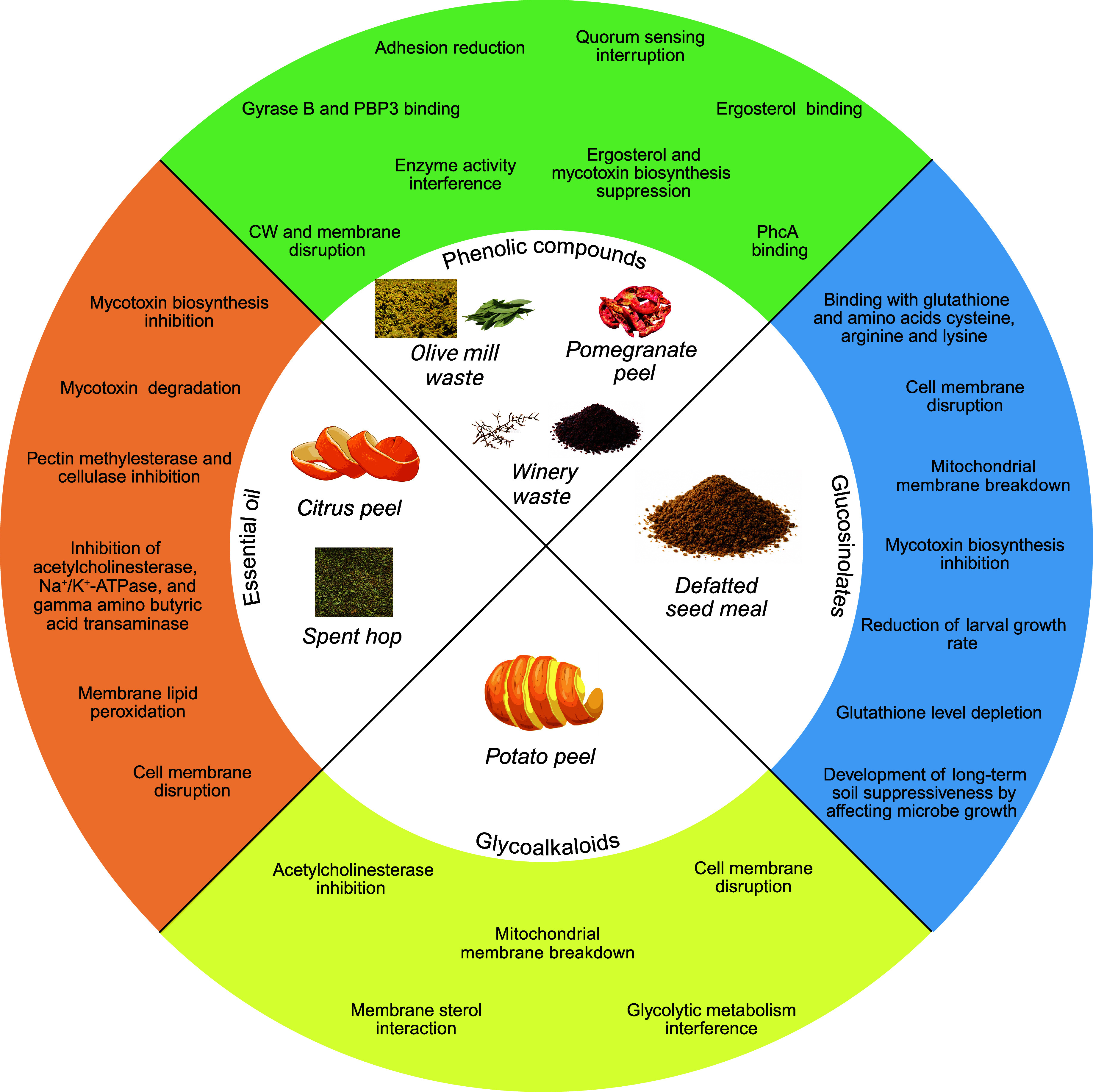
Donut chart of the antimicrobial and insecticidal mechanisms of action of agro-industrial byproduct extracts. The chart groups agro-industrial byproduct extracts according to the chemical nature of their bioactive compounds (inner sectors) and summarizes the main antimicrobial and insecticidal mechanisms of action reported for each group (outer ring).

### 4.1 Phenolic Compounds-Enriched Extracts from Agro-Industrial Byproducts

Many plant-derived agro-industrial byproducts are rich in PC spanning multiple chemical classes, including simple PCs, phenolic acids, flavonoids, coumarins, stilbenes, tannins, lignans, and lignins.^79^ They contribute to defense by reinforcing the CW via lignin/suberin deposition and by exerting antibacterial and antifungal effects.^80^

#### 4.1.1 Olive Mill Wastewater and Olive Pomace

Olive fruits are rich in PC, most of which (∼98%) partition into olive mill wastes during oil extraction.^81,82^ Early studies demonstrated that olive pomace fractions exert *in vitro* bactericidal activity against *Erwinia uredovora*, *Erwinia toletana*, *E. amylovora*, *P. savastanoi*, *P. syringae*, and *Clavibacter michiganensis*.^83^ Similarly, application of undiluted wastewater to potato dextrose agar medium markedly reduced the mycelial growth of several soil-borne phytopathogenic fungi, including *F. oxysporum*, *Pythium* spp., *Sclerotinia sclerotiorum*, *V. dahliae*, *Botrytis tulipae*, *A. niger*, and *Penicillium* spp.^84^ In pot trials, crude wastewater reduced crown gall symptoms and suppressed damping-off in tomato caused by *Rhizoctonia solani* and *F. solani*.^85,86^ Because raw wastes often require high application rates, extraction can reduce the dose needed.

Processing approaches have further enhanced the antimicrobial efficacy of olive-derived PCs. Water–ethanol olive pomace extracts inhibited mycelial growth of the phytopathogens *A. solani*, *B. cinerea*, and *F. culmorum*,^87^ *Cladosporium* sp., *A. alternata*, and *F. proliferatum*.^88^ The olive pomace liquid fraction subjected to tangential flow membrane filtration yielded a PCs mixture enriched in hydroxytyrosol (Htyr) that, at low concentrations, inhibited *in vitro* the growth of *P. syringae*, *P. carotovorum*, and notably *X. fastidiosa*. The same extract inhibited the growth of the fungi *C. graminicola*, *F. graminearum*, and *B. cinerea*.^89^ Comparable Htyr-rich extracts inhibited mycelium growth of *V. dahliae* and *A. solani* and exhibited antibacterial effects on *P. savastanoi* and *A. tumefaciens*, *P. syringae*, and *X. campestris*.^90,91^ Mechanistically, Htyr can disrupt CWs and membranes, increasing permeability, and may interfere with enzymes essential for DNA replication and CW integrity.^92^

The prevalence of Htyr in stored olive wastes is linked to oleuropein hydrolysis under acidic storage conditions.^93^ In fact, the major PCs detected in fresh olive fruit and leaves are the secoiridoid oleuropein.^94^ Also, oleuropein-enriched extracts showed antibacterial activity against *X. fastidiosa* ^95^ and counteracted *X. fastidiosa* proliferation in naturally infected olive trees through endotherapeutic administration.^96^ Oleuropein antimicrobial activity has been linked to modulation of morphogenesis, reduced surface adhesion, inhibition of pathogenicity-related enzymes, and interactions with membrane phosphatidylglycerol leading to pore formation and cell death.^97^ Other wastewater-associated PCs, such as catechol and methyl catechol, caffeic acid, and verbascoside, can inhibit *X. fastidiosa* growth.^95^ Overall, the antimicrobial efficacy of PC-rich extracts is likely due to the synergistic interplay among multiple constituents rather than the action of a single dominant compound. Interestingly, olive-derived PCs offer a promising and sustainable approach for controlling *X. fastidious*-associated olive quick decline syndrome and other plant diseases in Mediterranean agroecosystems.^89^

#### 4.1.2 Pomegranate Peel Fruit

The market demand for pomegranate fruit (species *P. granatum* L.) and derived products has increased greatly over the past decade, due to their health-promoting benefits.^98^ Industrial pomegranate juice production generates large amounts of peel waste, accounting for about 40% of the fresh fruit.^99^ Intriguingly, pomegranate peel is particularly rich in PCs, mainly hydrolyzable ellagitannins, anthocyanins, and flavonoids.^100^ Among them, punicalagin and its metabolite ellagic acid constitute the main bioactive components in extracts of pomegranate peel polyphenols (PPPs) associated with antifungal activity. Punicalagin-rich PPPs inhibited *in vitro* the growth of postharvest fungi such as *A. alternata*, *S. botryosum*, and *Fusarium* spp.,^101^ and controlled several postharvest diseases caused by *B. cinerea*, *M. laxa*, *P. digitatum*, *P. italicum*, and *P. expansum* on different fruits.^102^ Soil application of punicalagin- and ellagic acid-enriched PPPs reduced Fusarium wilt disease on tomato.^103^ These extracts are commonly obtained by using water or ethanol, enabling cost-effective and environmentally acceptable field applications. Indeed, PPPs sprays were effective against green and blue molds caused by *P. digitatum* and *P. italicum* in citrus orchards, outperforming the commercial fungicide Imazalil.^104^

Extraction solvent can influence the PPPs content and activity. Methanolic extracts often show higher antifungal efficacy than ethanolic or aqueous ones against *P. digitatum*, *P. italicum*, *R. stolonifer*, and *B. cinerea*,^105^ likely due to higher PCs content. However, the use of methanol on fruit commodities should be avoided due to the associated health risks.^106^ PPPs can induce marked hyphal abnormalities in several fungi,^105^ probably through punicalagin interaction with ergosterol, leading to membrane pore formation and cytoplasmic leakage.^102^ PPPs also inhibit enzymatic steps in ergosterol and aflatoxin biosynthesis in *A. flavus*, suggesting potential against mycotoxigenic fungi.^107^ However, antifungal activity is not always strictly correlated with punicalagin content, indicating a role for additional phenolics.^108^ PPPs antibacterial activity was associated with different PCs. Growth of *R. solanacearum* was inhibited by punicalagin-rich extracts.^109^ Ellagic was the main active component of PPPs extracts that successfully reduced the disease severity caused by *P. syringae* pv *tomato* on tomato plants.^110^ Extracts enriched in punicalin and gallic acid controlled black rot caused by *X. campestris* pv *campestris* on *B. rapa*.^111^ Gallic and ellagic acids interfere with bacterial motility and biofilm formation, disrupting quorum sensing, and PPPs may further compromise membrane integrity, promoting cellular damage.^109^ Punicalagin has also been proposed to inhibit virulence in *R. solanacearum* by targeting PhcA, a putative transcriptional regulator.

#### 4.1.3 Winery Wastes: Grape Pomace and Grape Stem

The plant *V. vinifera* is the most commonly cultivated species for wine production.^112^ The total global production of wine in 2024 was estimated at around 226 million hectoliters.^113^ About 30% of processed grapes are discarded as winery wastes, representing nearly 20 million tons per year.^114^ Winery wastes are a valuable source of PCs, since only 30–40% are extracted during vinification.^115,116^ Winery wastes mainly include grape stem (also referred to as stalk or rachis), grape pomace, wine lees, and wastewater. Grape stems are usually removed before the vinification steps due to their high content of proanthocyanidins, flavan-3-ols, and phenolic acids, which impart astringency and bitterness.^117–119^ Maceration of powdered grape stems in acetone, ethyl acetate, methanol, or ethanol yielded phenolic extracts active against *A. flavus*, *A. fumigatus*, *A. niger*, *P. chrysogenum*, *P. italicum*, and *P. expansum*.^120^ Ultrasonic extraction of powdered grape stems in 1:1 ethanol–water bath yielded an extract enriched in p-coumaric and ferulic acids with antifungal effects against *A. carbonarius*. Additionally, application of these extracts by microencapsulation reduced ochratoxin levels in fresh-cut fruit and in fruit juices.^121^ Grape pomace consists of grape skins, seeds, pulp, and yeast residues.^122^ Acid hydrolysates of grape skins and seeds inhibited *F. oxysporum* due to phenolic acids such as gallic, hydroxybenzoic, p-coumaric, and vanillic acids.^123^ Solvent extracts of red grape skins inhibited *P. chrysogenum*, *P. expansum*, *A. niger*, and *A. versicolor*, with methanol being the most effective solvent followed by acetone, ethanol, and water. Grape pomace hydroalcoholic extract, rich in the anthocyanin malvidin, inhibited the growth of the *P. syringae* pv *actinidiae* causing bacterial canker infection on leaves and trunks of kiwifruit trees.^124^ PCs were extracted from *V. vinifera* var. *Albariño* grape pomace using a patented hydro-organic solvent mixture, enriched in catechin, epicatechin, miquelianin, phloroglucinic acid, and procyanidin, inhibited the growth of the oomycete *P. cinnamomi*.^125^ A supercritical CO_2_ extract from red grape pomace of the Cabernet variety, rich in gallic acid, chlorogenic acid, kaempferol, ferulic acid, and quercetin, showed antifungal activity against *A. niger*.^126^ Ultrasound- or pressure-assisted extraction produced grape pomace extracts rich in anthocyanins (oenin, myrtillin, kuromanin) that reduced growth and mycotoxin production of *A. flavus*, *A. carbonarius*, and *F. graminearum*, particularly zearalenone and fumonisins.^127^ Similar effects were observed for white grape pomace extracts. These extracts also inhibited alternariol production by *A. alternata* without affecting fungal growth, indicating specific metabolic interference.^127^ PCs from grape wastes may limit mycotoxin accumulation through redox-mediated disruption of biosynthesis pathways.^121^ Their antimicrobial effects are likely linked to interactions of hydroxyl groups with microbial membranes, causing proton exchange, membrane destabilization, and cell lysis.^123^ However, the specific contribution of individual PCs present in grape waste extracts to these activities remains to be fully elucidated.

### 4.2 Steroidal Glycoalkaloids Extracts from Potato Peels

Potato (*S. tuberosum* L.) is the fourth major food crop in the world, after rice, wheat, and maize, with a production of around 383 million tons per year.^128^ Industrial processing generates large amounts of peel waste, accounting for 15–40% of tuber mass depending on peeling methods and totaling 70–140 thousand tons annually.^129^ Potato peels can be considered a great source of SG and PC, representing almost 50 and 70% of total alkaloids and PC in the whole tuber, respectively.^130^ These bioactive molecules are utilized by potato plants as part of their pest resistance mechanisms, providing a general protective effect against phytopathogens and pests.^131^ Larval mortality of the potato tuber moth (*Phthorimaea operculella*) correlates positively with peel concentrations of α-chaconine and the phenolics chlorogenic and caffeic acids^132^ which may act synergistically, as observed against *Mycosphaerella pinodes*, *A. alternata*, *Pyrenophora teres*, and *Pyrenophora tritici-repentis*. Pure form of SG naturally present in potato exhibited phytopesticide activities, such as solanidine to *Phytophthora infestans* ^133^ and α-chaconine and α-solanine to fungal phytopathogens *Alternaria brassicicola*, *Phoma medicaginis*, *R. solani* ^134^ and *Curvularia trifolii*, and insect pests *T. castaneum*, *S. oryzae*,^135^ *Trogoderma granarium*, and *Myzus persicae*. Notably, α-solanine and α-chaconine constitute >95% of total SGs in potato.^136^ Few studies have focused on SG extraction from potato peels. Zhang et al. (2023) showed that peel-derived SG inhibits colony growth, biomass, and spore germination of *F. solani* *in vitro*.^137^ In this study, SG were extracted by the acetic acid-ammonia precipitation method which could pose safety challenges in case of improper handling or exposure.^138^ Alternative approaches include ultrasound-assisted extraction with ethanol, which produced antifungal extracts protecting tubers against *F. sulphureum*.^139^ SG insecticidal activity is attributed to acetylcholinesterase inhibition and membrane disruption via sterol interactions.^133^ In fungi, SG penetrate cells, disrupt mitochondrial membranes, and alter enzyme activity and expression of glycolysis-related genes, reducing energy charge and pathogenicity.^137,140^ Owing to their hydrophobic steroidal core, SG is commonly dissolved in methanol prior to testing, raising environmental and health concerns. However, acidified ethanol-based solvents can yield aqueous SG formulations comparable to water/methanol extracts.^141^ α-solanine isolated from potato peels by silica gel chromatography and dissolved in acidic 80% ethanol showed strong antifungal activity against *B. cinerea*.^142^ Nevertheless, sustainable extraction strategies for agricultural use of potato peel SG remain underexplored and warrant further investigation.

### 4.3 Essential Oil-Enriched Extracts from Agro-Industrial Byproducts

EO are complex mixtures of numerous low-molecular-weight molecules, primarily composed of terpenoids and phenylpropanoids.^64^ Terpenoids derive from isoprene units, whereas phenylpropanoids originate from phenylalanine and tyrosine pathways.^143^ EO are volatile, soluble in organic solvents, and poorly soluble in water.^144^ They are produced as plant defense metabolites with insecticidal and antimicrobial properties and accumulate in specialized organs such as flowers, leaves, roots, bark, and fruits.^145^

#### 4.3.1 Citrus Peel Fruit

In *Citrus* fruits, EO accumulate mainly in peels (0.1–4.6% of dry weight) within oil glands of the flavedo.^146,147^ Citrus peel AIB can represent a valuable EO resource, as they are generated in high amounts during industrial orange juice production.^148^ Oranges account for 47.6% of global citrus production, followed by other economically important citrus fruits such as mandarins, lemons, limes, and grapefruits.^149^ Citrus EO are composed primarily of monoterpenes, with limonene being often the major compound, followed by citral and linalyl acetate, although composition varies with genotype and environment.^147^ EO are industrially extracted from citrus peel through cold pressing, a process in which the peel is mechanically ground and pressed to produce a watery emulsion.^150^ This emulsion is then centrifuged, and the supernatant is dehydrated using anhydrous Na_2_SO_4_ to recover EO. Limonene-rich EO from *C. aurantium* var. *dulcis* and *C. limon* reduced *C. okinawense* growth.^151^ EO from *C. limon*, *C. reticulata*, *C. paradisi*, and *C. sinensis* completely inhibited *A. niger*, *A. flavus*, *P. chrysogenum*, and *Penicilium verrucosum*.^152^ Vietnamese citrus peel EO inhibited *M. hiemalis*, *P. expansum*, and *F. proliferatum*, with lime oil fully inhibiting *M. hiemalis*.^153^ The antimicrobial activities of citrus EO extracted by cold pressing were also investigated for food preservation and safety. Vapor-phase application of mandarin EO rich in limonene reduced the mycelial growth of *P. chrysogenum* on wheat bread.^154^ Similarly, linalyl acetate-enriched EO from bitter orange (*C. aurantium*) peel exhibited antifungal activity in the vapor phase against *P. crustosum*, *P. citrinum*, and *P. expansum* on wheat bread and carrot.^155^ Liquid limonene-rich EO from sweet orange peel inhibited the growth of the cereal mycotoxigenic fungi *F. graminearum* and *F. culmorum* and reduced their mycotoxin accumulation in maize and wheat seeds.^156^ Although reduced mycotoxin levels may partly result from growth inhibition, citrus EO also appears to be capable of directly degrading mycotoxins. Limonene-rich EO from lemon, pink grapefruit, and white grapefruit (*C. grandis*) peels decreased deoxynivalenol concentrations,^157^ while citral-enriched lemon EO degraded fumonisin B1.^158^ Collectively, these findings highlight the potential of citrus EO to mitigate mycotoxin contamination during crop production, storage, and food or feed processing, although the underlying mechanisms require further investigation.

Hydrodistillation is an alternative method for citrus EO extraction, in which fresh or dried peels are exposed to boiling water, allowing volatile compounds to be transported with steam and recovered by condensation and phase separation.^159^ Limonene-rich EO obtained from *C. sinensis* peels by hydrodistillation completely inhibited mycelial growth and spore germination of several phytopathogens, including *A. alternata*, *A. mali*, *B. cinerea*, *C. cladosporioides*, *C. fulvum*, *P. chrysogenum*, *P. expansum*, *B. theobromae*, and *M. roridum*.^160^ Lower doses from sweet orange and pummelo (*C. maxima*) were effective against *A. terreus*, *A. alternata*, *F. oxysporum*, and *H. oryzae*.^161^ Hydrodistilled EO from *C. reticulata* (Shatangju) inhibited *P. expansum* and *P. sclerotiorum* at low dose, whereas a higher dose was required against *P. digitatum* and *P. italicum*,^162^ suggesting species-specific susceptibility. Consistently, EO from citrus species tolerant to *P. angolensis* showed stronger antifungal activity than that from susceptible species.^163^ Treatment of detached mandarin leaves with EO from tolerant varieties reduced the severity of the Alternaria brown spot disease caused by *A. alternata* f. sp. *citri*.^164^ Notably, limonene-rich EOs from the same citrus species may differ in activity, as observed for *C. sinensis* against *A. niger*, likely due to synergistic effects of minor components despite limonene being dominant.^165^ Their antifungal action is linked to membrane disruption driven by hydrophobic interactions, ROS accumulation, and lipid peroxidation (e.g., in *P. italicum*), and inhibition of fungal pectin methylesterase and cellulase.^165–167^ Limonene-rich EO also interferes with carbohydrate metabolism, reducing aflatoxin biosynthesis.^161^

Citrus EO further exhibits strong insecticidal activity via fumigant toxicity. Hydrodistilled peel EO from *C. aurantium*, *C. aurantifolia*, *C. limonium*, *C. latifolia*, *C. reticulata*, *C. sinensis*, and *C. paradisi* protected stored cowpea grains against *C. maculatus* ^168–170^ and reduced feeding damage by *Z. subfasciatus*, *S. zeamais*, *S. oryzae*, *T. castaneum*, *T. confusum*, and *R. dominica*.^171^ These effects are mainly attributed to limonene-mediated inhibition of acetylcholinesterase, Na^+^/K^+^- adenosine 5′-triphosphatase, and γ-aminobutyric acid transaminase.^172^ Antibacterial activity has been less explored but appears composition-dependent: *C. aurantium* peel EO rich in linalyl acetate was bactericidal to *X. citri* subsp. *citri*.^173^ As observed for fungi, the antibacterial activity of EO is mainly related to the ability of terpenes to alter membrane permeability and interfere with extracellular and intracellular enzymatic systems, ultimately leading to cell death.^152^ Notably, hydrodistillation also generates an aqueous byproduct, known as hydrolate, containing approximately 0.1% EO compounds.^174^ Linalool-rich hydrolates were obtained from *C. aurantium* var. *amara* peels, which exhibited antibacterial activity against *E. amylovora*, *P. savastanoi* subsp. *Savastanoi*, *X. vesicatoria*, and *A. vitis*.^175^ Apparently, the hydrophilic nature of hydrolates may enhance their antibacterial activity compared to lipophilic EO, making cell membranes more fluid and permeable to EO, particularly against Gram-negative bacteria with outer membranes rich in lipopolysaccharide-bound fatty acids.^175^

#### 4.3.2 Brewing Industry Wastes: Spent Hops

The global brewing industry is one of the major economic forces, accounting for approximately 0.8% of the global economy,^176^ and generates large amounts of solid residues, including spent grain, spent yeast, and spent hops (*H. lupulus*, trub), exceeding 20 million tons annually.^177^ Spent hops are a promising source of EO for crop protection. EO obtained by hydrodistillation inhibited the biosynthesis of zearalenone, ochratoxin A, and tenuazonic acid without significantly affecting the growth of their fungal producers (*F. graminearum*, *A. carbonarius*, and *A. alternata*).^127^ These effects were associated with terpene components such as myrcene, α- and β-caryophyllene, cadinene, and humulene epoxide. However, the underlying mechanisms remain to be clarified. Moreover, other brewing byproducts deserve further investigation for upcycling in sustainable agriculture, given their potential content of valuable bioactive compounds.^178^

### 4.4 Glucosinolate-Enriched Extracts from Defatted Seed Meal

Brassicaceae oilseed species is the third most important oil crop, after palm and soybean, contributing approximately 15% of the global vegetable oil production.^179^ After oil extraction, defatted seed meal is the main byproduct, accounting for ∼50% of seed mass and ∼49 million tons annually.^180^ Defatted seed meal contains high levels of GLS, translocated in the seeds during ripening and involved in Brassicaceae defense.^181^ GLS become biologically active after hydrolysis by myrosinase, forming glucosinolate hydrolysis products, whose nature depends on side-chain structure, associated proteins, and reaction conditions.^182^ The volatility of certain hydrolysis products enables their application in pest and phytopathogen control via biofumigation. Treatments based on allyl isothiocyanate released from defatted seed meal of *B. carinata* or on butenyl isothiocyanate from *B. rapa* seed meal completely inhibited *M. laxa* on stone fruits,^183^ while allyl isothiocyanate from *B. juncea* defatted seed meal reduced *P. expansum* growth on pears.^184^ Strawberry exposure to allyl isothiocyanate released from *B. carinata* seed meal lowered *B. cinerea* incidence.^185^ Interestingly, a sealed environment is not required for volatile products to exhibit their antimicrobial properties, paving the way for the use of defatted Brassica seed meal on crops cultivated in greenhouse and open field conditions. Powdered defatted seed meal from *B. carinata*, formulated with vegetable or mineral oils, reduced powdery mildew severity on sugar beet (*E. betae*) and on muskmelon (*E. cichoracearum*).^186^ Because of the high volatility of certain glucosinolate hydrolysis products, oils are incorporated to improve their stability.^187^ Within a circular-economy framework, Brassica oil represents a sustainable formulation component. Field sprays of Brassica defatted seed meal-oil formulations controlled *T. urticae* on eggplant^188^ and *P. xanthii* ^189^ on melon, with the higher dose performing comparably to penconazole (Topas). A formulation containing *B. carinata* oil combined with meal from the same species and gum arabic also outperformed dodine against olive leaf spot (*F. oleagineum*).^190^ The GLS-containing Brassica seed meal can promote the development of suppressive soil that inhibits soil-borne phytopathogens.^191^ Seed meals rich in GLS from *B. juncea* or *Sinapis alba* reduced Fusarium wilt on chili pepper.^192^ Isothiocyanates diffuse across membranes and react with glutathione and amino acids, causing structural damage and oxidative stress.^193^ In fungi, exposure to isothiocyanates can induce membrane breakdown, reduced respiration, and mitochondrial depolarization, while also inhibiting mycotoxin biosynthesis.^194^ Ingestion of GLS by insects can reduce larval growth rate, leading to longer development times, fewer generations per season, and prolonged exposure to predators, partly through glutathione depletion.^195^ Soil amendment with seed meal can further support long-term disease suppression by enriching *Streptomyces* spp.^196^ Furthermore, soil microbes can oxidize the ammonium incorporated in seed meal, leading to nitric oxide that may contribute to the activation of plant systemic defenses.^197^ This explains reduced *R. solani* infection in apple seedlings grown in seed meal-amended soil independently of GLS hydrolysis products.^198^ In apple orchards, soil treatments based on defatted seed meal from *B. juncea*, *B. napus*, or *S. alba* provided replant performance equal or superior to chemical fumigation with Telone-C17.^199,200^

## 5 Agro-Industrial Byproducts as Source of Plant Immunity Elicitors

AIB represents a valuable reservoir of bioactive molecules that can act as elicitors of plant immunity. Processing residues from plant- and microbe-based biomasses can release diverse compounds, including CW-derived oligosaccharides, microbial and plant proteins, and phenolic constituents. These molecules function as DAMPs, MAMPs, or phytocytokines capable of triggering immune responses in plants through the activation of PTI signaling. Overviews of AIB extracts acting as inducers of plant defense responses and their action mechanisms are summarized in Table 3.

**Table 3:** Agro-Industrial Byproducts Extracts Acting as Plant Defense Elicitors^a^

**byproducts**	**species**	**extraction**	**fractionation**	**composition**	**mechanisms of action**	**pathosystems**	**refs**
olive pomace	*O. europaea*	hydroalcholic	filtration	oligogalacturonides, arabinooligosaccharides	activation of MAPK-mediated signaling and WRKY-dependent transcriptional regulation leading to induction of phytoalexin biosynthesis and defense-related genes	*B. cinerea*/*Arabidopsis thaliana*	^202^
		N.R./	tangential flow membrane filtration	77% Htyr, 18.5% tyrosol	induction of ROS burst and MAPK activation associated with transcriptional activation of pathogen-responsive genes	*P. syringae*/*A. thaliana*	^89^
olive mill digestate		ultrasonic/organic solvent	centrifugation	elongation factor Tu, Endo-1,4-B-xylanase, flagellin, histidine kinase, pectate lyase, golven 1–2	activation of pattern-triggered immunity through ROS production, MAPK cascades and expression of genes involved in antimicrobial secondary metabolism	*P. syringae*/*Solanum lycopersicum*	^217^
agar alkaline extraction waste	*Gelidium sesquipedale*	organic solvents	N.R.	glycerol-galactosides	induction of pathogenesis-related proteins involved in cell wall reinforcement and antimicrobial activity	*B. cinerea*/*S. Lycopersicum*	^213^
						*Plasmopara viticola*/*V. vinifera*	
molasse	*β vulgaris*	organic solvents	functionalization	chemically modified pyroglutamic acid	activation of phenylpropanoid metabolism and oxidative enzymes associated with defense-related gene expression	*Zymoseptoria tritici*/*Triticum aestivum*	^219^
pomegranate peel fruit	*P. granatum*	hydroalcholic	filtration	punicalagin, gallic acid, ellagic acid, granatin	activation of MAPK signaling and phenylpropanoid biosynthesis genes involved in phytoalexin production and oxidative defense	*P. digitatum*/*Citrus × limon*, *P. italicum*/*Citrus × limon*, *P. digitatum*/*V. vinifera*, *P. italicum*/*V. vinifera*	^229^
					N.R.	*Colletotrichum a* *cutatum*/*O. europaea*, *Venturia oleaginea*/*O. europaea*	^232,233^
		organic solvents	filtration	N.R.	modulation of plant defense responses potentially linked to priming of immune-related gene expression	*P. carotovorum*/*S. tuberosum*	^231^
citrus fruit peel	*C. aurantium*	hydrodistillation	N.R.	linalool	N.R.	*X. vesicatoria*/*S. lycopersicum*	^175^

a N.R.: not reported, ×: interspecific hybridization.

A model illustrating the perception of biomolecules in AIB extracts by specific receptors and the activation of plant immune signaling pathways is shown in Figure 2.

**Figure 2:**
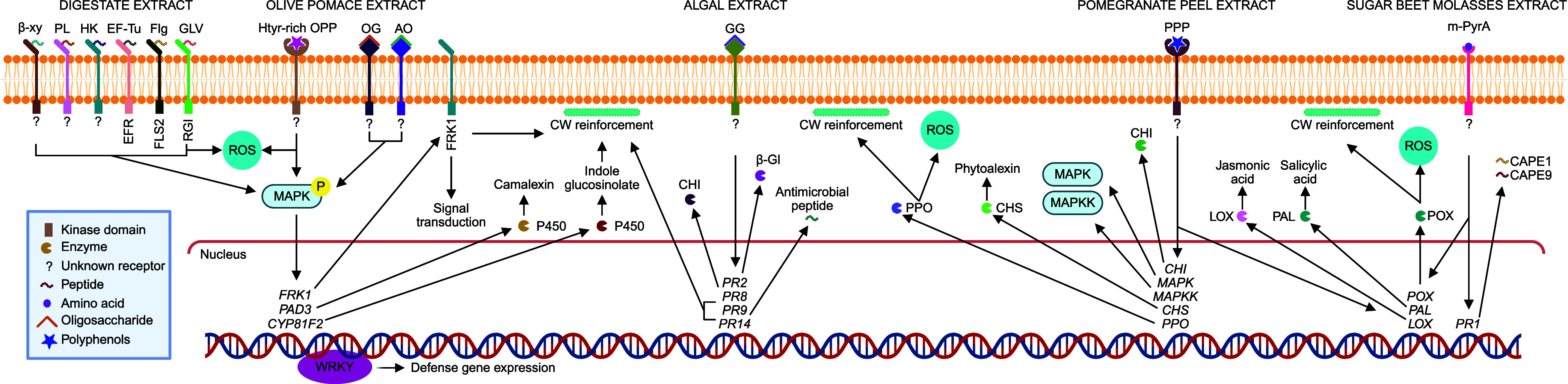
Model of biomolecule recognition from agro-industrial extracts leading to the activation of plant immune signaling pathways. Biomolecules present in agro-industrial byproduct extracts, including peptides, amino acids, oligosaccharides, and polyphenols, can be perceived at the plant cell surface by known or unidentified pattern-recognition receptors (PRRs). Digestate-derived peptides such as elongation factor Tu (EF-Tu), Flagellin (Flg), and Golven (GLV) are recognized by EFR, FLS2, and RGI receptors, whereas receptors for peptides derived from 1,4-β-xylanase, pectate lyase, and histidine kinase remain unknown. Ligand perception can induce reactive oxygen species (ROS) production and activate MAPK/MAPKK cascades (MAPK3, MAPK4, MAPK6, and MAPK11), leading to WRKY-dependent transcriptional reprogramming (WRKY33, WRKY53) and induction of defense marker genes such as FRK1. Downstream responses can include the activation of cytochrome P450–dependent pathways involved in phytoalexin and indole glucosinolate biosynthesis (*PAD3*, *CYP81F2*), associated with CW reinforcement. Similar signaling pathways can be activated by hydroxytyrosol-rich olive pomace polyphenols (Htyr-rich OPP), oligogalacturonides (OG), and arabino-oligosaccharides (AO). Glycerol-galactosides can induce pathogenesis-related proteins (PR2, PR8, PR9, PR14) involved in antimicrobial responses and lignification. Polyphenols from pomegranate peel and modified pyroglutamic acid extracted from sugar beet molasse can activate MAPK-dependent signaling and enzymes related to phenylpropanoid metabolism, oxidative defense, and hormone-associated pathways (PAL, POX, LOX, PR1). PR1 can be proteolytically processed to release immunogenic phytocytokines CAPE1 and CAPE9. The mechanisms illustrated are based on information obtained from individual elicitors; in complex extracts, multiple biomolecules may lead to the simultaneous activation of different immune signaling pathways.

### 5.1 Agro-Industrial Byproducts are Rich in Glycan-Derived Elicitors of Plant Immunity

DAMPs include CW-derived oligosaccharides released during infection or tissue damage.^16,201^ In olive oil mills, CW fragments can originate from fruit ripening and milling.^202^ A pectic mixture of OG (degree of polymerization 1–30) and arabino-oligosaccharides (degree of polymerization 2–8), extracted from the liquid fraction of olive pomace using aqueous ethanol, activated mitogen-activated protein kinase-3 (MAPK3) and MAPK6 phosphorylation and induced defense genes *PHYTOALEXIN DEFICIENT 3* (*PAD3*), *CYTOCHROME P450, FAMILY 81* (*CYP81F2*), *FLG22-INDUCED RECEPTOR-LIKE KINASE 1* (*FRK1*), *WRKY DNA-BINDING PROTEIN 53* (*WRKY53*), and *WRKY33* in *A. thaliana*.^203,204^ These genes are involved in camalexin biosynthesis, callose deposition, and defense signaling.^205,206^ Foliar pretreatment with olive pomace-extracted pectic oligosaccharides enhanced resistance to *P. syringae* and *B. cinerea* in *A. thaliana* and tomato, reducing bacterial populations and disease symptoms.^202^ Although OG has been shown to bind Wall-Associated Kinase (WAK) receptors in *A. thaliana*, the full set of receptors involved in OG perception and downstream immunity activation remains to be elucidated.^207^ Moreover, the degree of OG methylesterification can contribute to elicitor activity.^208^ Marine macroalgae CWs represent an additional source of glycan-derived DAMPs.^209^ Marine macroalgae belong to three taxonomic groups such as phylum Chlorophyta or green macroalgae, phylum Heterokontophyta or brown macroalgae, and phylum Rhodophyta or red macroalgae, each exhibiting distinct CW compositions.^210^ Red macroalgae CWs, rich in galactans such as carrageenans and agarans,^211^ generate alkaline extraction wastes during agar production. These wastes contain galactan-derived oligosaccharides that, when applied to tomato plants, induced the expression of pathogenesis-related proteins 2 (PR2), encoding a β-1,3-glucanase, and PR8, encoding a type III chitinase, both enzymes involved in pathogen CW hydrolysis, and enhanced resistance to *B. cinerea*.^212,213^ Notably, in *B. cinerea*-inoculated tomato plants, agar extraction wastes also induced the expression of CW reinforcement-related genes, including *PR9* and *PR14*, whereas this response was not observed in noninoculated plants, indicating that pathogen presence is required for full elicitor activity.^213^ In addition, *PR14* encodes antimicrobial peptides that promote pathogen membrane permeabilization.^214^ Field applications of these extracts further increased grapevine resistance to *P. viticola*, one of the most damaging pathogens in viticulture.^213^

### 5.2 Protein/Peptide-Derived Elicitors of Plant Immunity

Olive pomace is increasingly valorized in biorefineries for anaerobic digestion,^215^ generating digestates rich in partially degraded organic matter and microbial biomass.^216^ Microbial biomass can be recovered from the liquid fraction of olive mill digestate and subjected to mechanical processing steps, including sedimentation, centrifugation, and sonication, to extract a protein mixture containing bacterial and fungal MAMP molecules such as EF-Tu, endo-1,4-β-xylanase, Flg, histidine kinase, and pectate lyase.^217^ The mechanical processing steps also involved plant residues, as evidenced by the identification of a plant phytocytokine homologue to Golven (GLV) peptides in the digestate protein extract. These molecules are known to be processed by plant apoplastic proteases, releasing peptide elicitors that are recognized by specific surface receptors to activate plant immune defenses.^29,32^ Indeed, olive mill digestate protein extracts activated PTI signaling pathways such as a transient burst of H_2_O_2_, phosphorylation of MAPK6, MAPK3, and MAPK4/11, and upregulation of defense genes *CYP81F2*, *FRK1*, and *WRKY53*.^217^ The immune system activation conferred disease resistance in *A. thaliana* and tomato plants against *B. cinerea* and *P. syringae*.

Chemical modification of AIB-derived molecules can further enhance the elicitor activity. Molasses is a residual raw juice obtained during sugar beet processing for sucrose extraction from the roots.^218^ Molasses comprises approximately 30% amino acids, with glutamine and glutamic acid together constituting over 50% of total sugar beet amino acids.^219^ Thermal degradation of glutamine and glutamic acid leads to the formation of pyroglutamic acid^220^ that is detected in molasses during sugar beet root processing and/or storage. Pyroglutamic acid recovered from molasses can be functionalized via green chemistry approaches, yielding compounds that induce resistance in wheat against *Z. tritici* through activation lipoxygenases (LOX), phenylalanine ammonia-lyases (PAL), peroxidases (POX), and PR1.^219^ These enzymes regulate major defense pathways, with LOX and PAL governing jasmonic acid- and salicylic acid-mediated signaling, POXs promoting ROS production and CW reinforcement.^221^ Recently, PR1 was reported to be proteolytically cleaved to release the phytocytokine CAP-derived peptides, CAPE1 and CAPE9, that activate systemic immunity in *A. thaliana* and tomato, respectively.^222,223^

### 5.3 Phenolic Compounds and Essential Oils are Elicitors of Plant Immunity

Beyond antimicrobial activity, PC act as signaling molecules in plant defense.^224,225^ PC can be recovered from olive mill wastes using tangential flow membrane filtration coupled with reverse osmosis.^226^ Exogenous application of Htyr-rich phenolic mixtures extracted from olive pomace induced PTI hallmarks, including a transient H_2_O_2_ burst, MAPK6 phosphorylation, and upregulation of *CYP81F2*, *FRK1*, and WRKY53.^89^ Pretreatment with olive pomace-extracted PC primed *A. thaliana* and tomato defenses against *B. cinerea* and *P. syringae*, while also protecting potato tubers from *P. carotovorum* soft rot,^227^ and suppressing tomato wilt caused by *V. dahliae*.^228^ These disease-inhibiting effects likely result from combined antimicrobial and immune-stimulating activities.

Hydroalcoholic PPP extracts activated defense responses in citrus fruits inoculated with *P. digitatum* and *P. italicum*, achieving complete decay reduction without direct pathogen contact.^229^ This effect was supported by transcriptomic analyses of PPP-treated fruits, which revealed the induction of multiple defense-related genes, including those encoding glutathione S-transferase, a key enzyme involved in detoxifying lipid hydroperoxides generated during pathogen-induced membrane peroxidation.^230^ Consistently, PPP treatments reduced *P. digitatum* and *P. italicum* growth in grapefruit through ROS accumulation and overexpression of genes encoding MAPK, MAPK kinase (MAPKK), PAL, chalcone synthase, and Chitinase, which are associated with defense signaling, phytoalexin biosynthesis, and fungal CW degradation.^229^

In potato plants, PPP treatment increased the total PC content and induced systemic resistance, associated with the upregulation of defense-related enzymes such as POX, PAL, and polyphenol oxidase (PPO), ultimately protecting seed tubers against *P. carotovorum* subsp. *carotovorum*.^231^ Similarly, the ability of hydroalcoholic PPP extracts to activate host resistance responses likely plays a major role in controlling olive anthracnose and olive leaf spot caused by *C. acutatum* and *V. oleaginea*, respectively, in orchards with a high incidence of latent infections.^232,233^ Although a direct antifungal effect on colonizing fungi cannot be excluded, there is currently no evidence supporting the penetration of PPP and diffusion within host tissues. Notably, field trials demonstrated that PPP treatments were significantly more effective than copper-based products and the commercial fungicide Flint Max in controlling these olive diseases.^232,233^

EO have also been reported to activate plant defense responses.^234^ In particular, tomato plants were pretreated with *C. aurantium* var. *amara* hydrolate enriched in linalool, obtained by fruit peel hydrodistillation, showed a reduction in bacterial leaf spot symptoms caused by *X. vesicatoria*.^175^ However, the molecular mechanisms underlying resistance induction by citrus peel EO remain to be clarified.

## 6 Perspectives and Future Directions

The use of biopesticides and bioelicitors to protect plants from pests and phytopathogens represents a promising and ecosustainable strategy to reduce or replace the use of synthetic pesticides in agriculture. Given their broad range of activities and high efficacy, bioactive molecules recovered from AIB have potential applications beyond organic farming, extending to conventional and integrated farming systems. The wide availability of AIB, together with its demonstrated bioactivity, supports the development of novel phytochemical-based commercial formulations. Sustainable extraction and separation technologies, coupled with appropriate solvent selection, offer a practical route to recover high-value bioactive molecules and convert residues into standardized ingredients for biopesticide and plant elicitor formulations. Genetic engineering and breeding approaches could further enhance the content and composition of bioactive molecules in AIB, enabling the development of industrially relevant genotypes enriched with antimicrobial compounds and elicitors. Nevertheless, despite compelling laboratory and small-scale field evidence, large-scale implementation remains limited. Future studies should systematically quantify bioactive compound levels in raw AIB data that are currently scarce and highly variable but essential for process standardization, scalability, and industrial deployment. Further research should focus on optimizing extraction and formulation strategies, elucidating molecular and epigenetic mechanisms of action, and conducting comprehensive agronomic trials to assess crop specificity and compatibility with integrated pest management.

From an economic perspective, process costs are largely driven by raw material logistics, extraction yield, solvent recovery, energy consumption, and downstream purification and vary according to the technologies and target compounds employed. Although advanced green extraction and membrane-based separation processes require higher initial investments, they may reduce operating costs through lower solvent consumption, shorter processing times, and improved product standardization. Regulatory approval remains a major bottleneck as AIB-derived plant protection products must comply with pesticide or biostimulant regulations, including safety, efficacy, and environmental risk assessments, which significantly affect time-to-market and development costs. Finally, comprehensive technoeconomic and life cycle assessment studies are still lacking for most AIB-based value chains and are crucial for evaluating feasibility, sustainability, and scale-up potential. Addressing these challenges will be essential to advance circular economy principles and promote resilient, eco-friendly agricultural systems.

## Abbreviations

AIB: agro-industrial byproducts
*CYP81F2*: cytochrome P450, family 81
CW: cell wall
CWDE: cell wall-degrading enzymes
DAMP: damage-associated molecular patterns
EF-Tu: elongation factor Tu
EFR: elongation factor Tu receptor
EO: essential oils
Flg: flagellin
FLS2: flagellin sensitive 2
FRK1: FLG22-induced receptor-like kinase 1
GLS: glucosinolates
GLV: golven
HG: homogalacturonan
Htyr: hydroxytyrosol
Htyr-rich OPP: hydroxytyrosol-rich olive pomace polyphenols
LOX: lipoxygenase
MAMP: microbe-associated molecular patterns
MAPK: mitogen-activated protein kinase
MAPKK: mitogen-activated protein kinase kinase
OG: oligogalacturonides
OPP: olive pomace polyphenols
PAD3: phytoalexin deficient 3
PAL: phenylalanine ammonia-lyase
PC: phenolic compounds
PL: pectate lyase
PPP: pomegranate peel polyphenols
PPO: polyphenol oxidase
PR: pathogenesis related protein
PRR: pattern recognition receptor
PTI: pattern-triggered immunity
POX: peroxidase
RG: rhamnogalacturonan
RGF1: root meristem growth factor 1
RGI: RGF1 insenitive
ROS: reactive oxygen species
SG: steroidal glycoalkaloids
WAK: wall-associated kinase
WRKY: WRKY DNA-binding protein
